# Nebulization and *In Vitro* Upper Airway Deposition of Liposomal Carrier Systems

**DOI:** 10.1021/acs.molpharmaceut.3c01146

**Published:** 2024-03-11

**Authors:** Ondrej Mišík, Jana Kejíková, Ondřej Cejpek, Milan Malý, Adam Jugl, Miloslav Bělka, Filip Mravec, František Lízal

**Affiliations:** 1 Department of Thermodynamics and Environmental Engineering, Faculty of Mechanical Engineering, Brno University of Technology, Technicka 2896/2, 616 69 Brno, Czech Republic; 2 Institute of Physical and Applied Chemistry, Faculty of Chemistry, Brno University of Technology, Purkyňova 464/118, Královo Pole, 612 00 Brno, Czech Republic

**Keywords:** liposome, aerosol, particle size, nebulizer, pulmonary drug delivery, inhalation, deposition

## Abstract

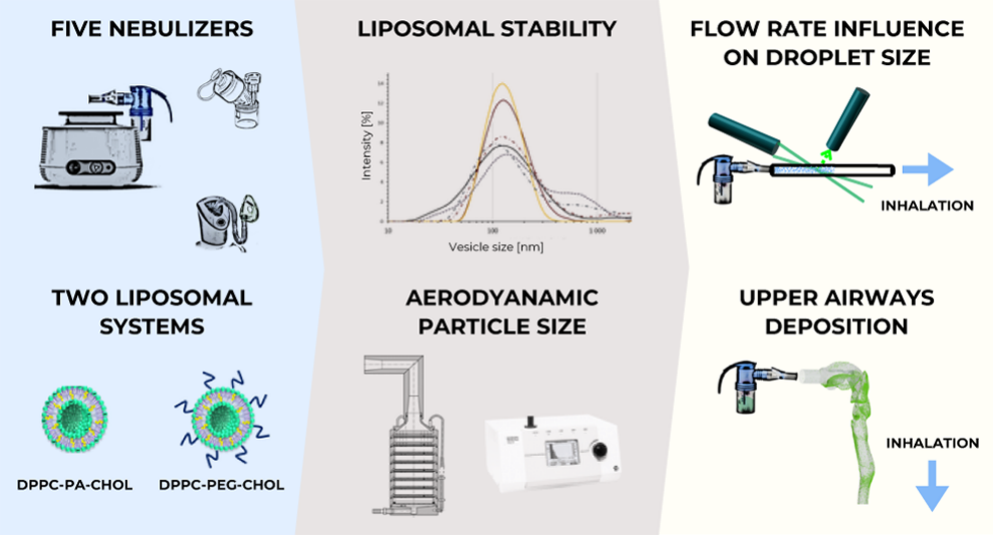

Liposomal carrier systems have emerged as a promising technology for pulmonary drug delivery. This study focuses on two selected liposomal systems, namely, dipalmitoylphosphatidylcholine stabilized by phosphatidic acid and cholesterol (DPPC-PA-Chol) and dipalmitoylphosphatidylcholine stabilized by polyethylene glycol and cholesterol (DPPC-PEG-Chol). First, the research investigates the stability of these liposomal systems during the atomization process using different kinds of nebulizers (air-jet, vibrating mesh, and ultrasonic). The study further explores the aerodynamic particle size distribution of the aerosol generated by the nebulizers. The nebulizer that demonstrated optimal stability and particle size was selected for more detailed investigation, including Andersen cascade impactor measurements, an assessment of the influence of flow rate and breathing profiles on aerosol particle size, and an *in vitro* deposition study on a realistic replica of the upper airways. The most suitable combination of a nebulizer and liposomal system was DPPC-PA-Chol nebulized by a Pari LC Sprint Star in terms of stability and particle size. The influence of the inspiration flow rate on the particle size was not very strong but was not negligible either (decrease of *D*_*v*50_ by 1.34 μm with the flow rate increase from 8 to 60 L/min). A similar effect was observed for realistic transient inhalation. According to the *in vitro* deposition measurement, approximately 90% and 70% of the aerosol penetrated downstream of the trachea using the stationary flow rate and the realistic breathing profile, respectively. These data provide an image of the potential applicability of liposomal carrier systems for nebulizer therapy. Regional lung drug deposition is patient-specific; therefore, deposition results might vary for different airway geometries. However, deposition measurement with realistic boundary conditions (airway geometry, breathing profile) brings a more realistic image of the drug delivery by the selected technology. Our results show how much data from cascade impactor testing or estimates from the fine fraction concept differ from those of a more realistic case.

## 1 Introduction

Particle inhalation is a local type of drug administration that could be used with benefits in the treatment of diseases such as cystic fibrosis, asthma, chronic pulmonary infections, pneumonia, chronic obstructive pulmonary disease, lung cancer, or COVID-19-associated pulmonary embolism^1−3^ but also in the treatment of systemic diseases such as Parkinson disease, migraine, diabetes, and others.^4^ The advantages of the local administration of drugs to the lungs are the mitigation of side effects and the possibility of higher dosages. One of the disadvantages of inhalation is the removal of the drug by pulmonary defense mechanisms and subsequently more frequent dosing. A proper carrier can overcome this disadvantage and enhance the treatment selectivity.^2,5^ One of the potential carriers is liposomes.

Liposomal carriers are promising means of drug delivery systems in various applications, such as anticancer,^6−9^ antifungal,^10^ anti-inflammatory treatment,^10^ gene therapy,^11^ diagnostics,^10^ and others. Liposomes are spherical vesicles that allow the incorporation of an active pharmaceutical ingredient (API) with different physicochemical properties.^12,13^ Their composition ensures biocompatibility, biodegradability, and safe use of liposomes not only for inhalation administration^3,14,15^ but also to actively target drug delivery systems to achieve a higher concentration of drugs inside the cells, decrease toxicity, and increase treatment effectiveness by adding ligands on the surface of liposomes.^16^

Liposomes need to be delivered to the lungs using a suitable instrument. There are four available types of inhaler technologies: dry powder inhalers, pressurized metered-dose inhalers, soft mist inhalers, and nebulizers. Most liposomal formulations are developed for nebulizer aerosolization.^5,17,18^

**Figure 1:**
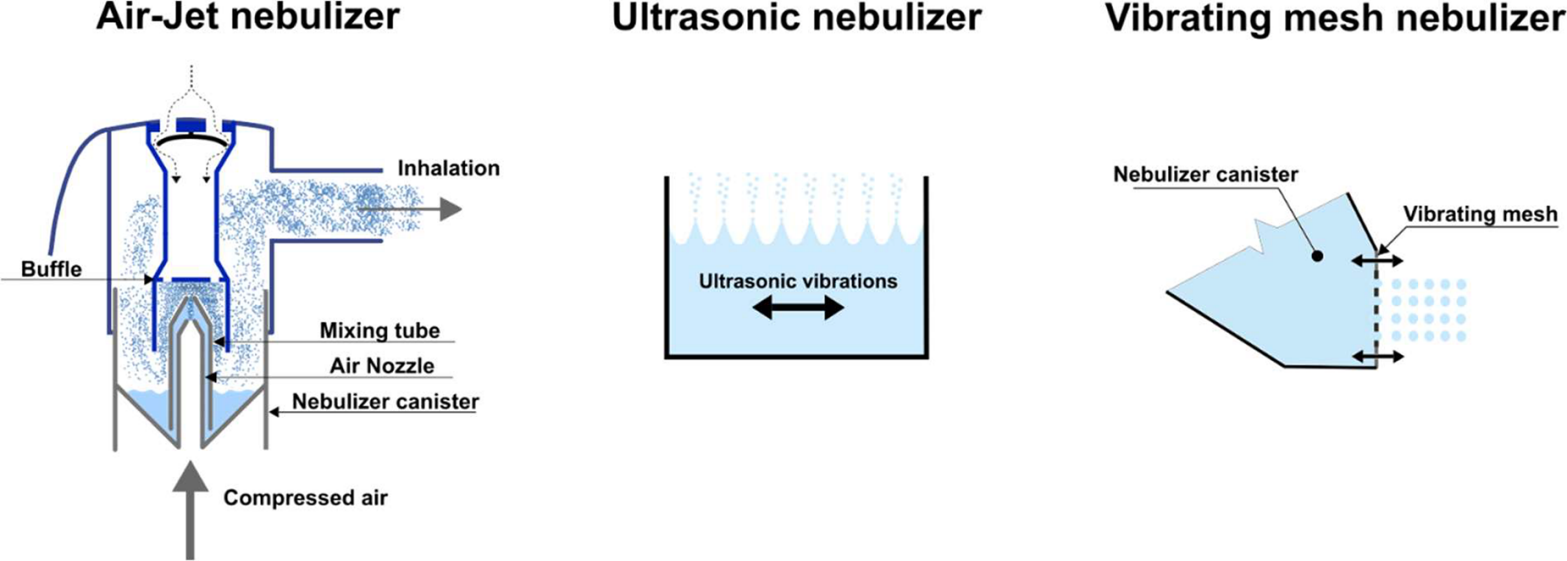
Principles of nebulizer technologies.

Nebulizers atomize the liquid formulation into inhalable particles.^19^ Compared with the other technologies, there is no need for complicated training since aerosol inhalation is performed by normal tidal breathing.^20,21^ There are three types of nebulizer technology: air-jet nebulizers (AJN), ultrasonic nebulizers (USN), and vibrating mesh nebulizers (VMN) *(*Figure 1*).* AJNs use compressed air for the atomization of the liquid. The air is driven through a nozzle placed in a mixing tube. The bottom of the tube is located in the nebulizer canister, which contained the liquid formulation. As the airstream is accelerated through the nozzle, the Venturi effect creates a negative pressure in the tube. Therefore, the liquid formulation is sucked through the tube into the airstream and atomized into airborne droplets by shear forces.^19,22^ In the case of USNs, the liquid formulation is placed in a container located in a water bath. A piezoelectric speaker creates ultrasonic vibrations and is placed at the bottom of the water bath. High-frequency vibrations lead to the formation of droplets on the surface of the formulation. However, this technology heats the formulation during nebulization, making it unsuitable for thermolabile compounds.^19,22^ VMNs contain a plate with many orifices the size of a few micrometers. This mesh vibrates at a high frequency and breaks off airborne droplets from the formulation upon contact with the liquid. Such devices are becoming the first choice technology.^19,22,23^

For a successful drug delivery of liposomal formulation, the vesicles must survive the atomization process and reach the target site of the airways. The most suitable nebulizers seem to be the air-jet and vibrating mesh.^9^ The ultrasonic nebulizers cause destabilization and disruption of the liposome structure, which leads to the release of encapsulated drugs due to heating by ultrasonic waves.^5^ In usual practice, various stabilizing agents are added to the vesicle formula to reduce the possibility of liposome disruption, e.g., cholesterol, polyethylene glycol, chitosan, or phosphatidic acid.^5,24−26^

Regional airway deposition of aerosol particles depends on several variables such as particle size, particle density, porosity, shape, velocity, and inspiration flow rate, breathing profile, and airway geometry. The aerodynamic diameter is used for particle size description concerning the aerodynamic behavior of aerosols. The aerodynamic diameter is the diameter of an equivalent spherical particle with a 1000 kg/m^3^ density and the same settling velocity as that of the investigated particle. It is used to predict aerosol deposition within the respiratory tract and can be measured with several devices. The *European Pharmacopoeia*(27) prescribes only cascade impactors and impingers that evaluate aerodynamic particle size by separating the aerosol particle size distribution (APSD) into several fractions by inertial impaction. Although the aerodynamic particle size is a crucial parameter for investigating deposition in the respiratory tract, the airway inspiration profile and geometry also play a significant role. A common assumption in the particle engineering literature is that aerosol particles should be designed in the 1–5 μm size range (fine particle fraction) to ensure successful pulmonary drug delivery.^28−31^ However, the dependence of regional deposition and particle size of the aerosol emitted from the inhaler is not clearly and precisely determined,^32^ and the studies which compared the FPF (fine particle fraction) of dry powder inhalers with *in vivo* lung deposition pointed out that the FPF characterized as particles between 1–5 μm overestimated the whole lung deposition^33,34^ The FPF and *in vivo* lung deposition correlation is expected to differ in the case of nebulizers used with normal tidal breathing at lower flow rates.^35^ During rapid, deep inhalation (metered dose inhalers and dry powder inhalers), the particles have a significantly higher Stokes number (*Stk*), resulting in a higher probability of wall deposition.

Current measuring techniques (impactors or impingers) prescribed for inhaler testing consider only inertial impaction as the acting deposition mechanism. However, a few other mechanisms also affect respiratory deposition.^36^ Besides the inertial impaction, gravitational sedimentation and Brownian diffusion may also play a significant role. Gravitational sedimentation dominates mainly in areas with low air velocity.^37^ Therefore, it is crucial mainly in the peripheral lung region, where the air velocity is low due to the sequential branching system of airways, but according to an experimental study,^38^ its influence is significant. The third important mechanism for respiratory deposition is Brownian diffusion. It occurs when the particles are small enough to be affected by the collisions with the surrounding gas molecules. This mechanism becomes dominant for particles around 0.3 μm, and its effect increases with a decreasing particle size. Deposition by this mechanism is effective in all airway regions.^36^

According to the studies above, the *in vitro* predictions overestimated regional deposition compared to the *in vivo* data. This could have been caused by several idealizations employed in inhaler testing methods, such as only one acting deposition mechanism, stationary inspiration flow rate of defined values, and idealized geometry. Therefore, the deposition in cascade impactors cannot be considered an analog of deposition in the respiratory tract. Also, as noted above, the concept of FPF as a predictor of potentially respirable aerosol particles does not consider the effect of the inhalation flow rate or airway geometry.

Hence, the current methods are prescribed by the *European Pharmacopoeia* do not provide sufficient information about the inhaled drug behavior in human lungs^39^ as they were primarily created for product development and quality control. The regional aerosol lung deposition is patient-specific due to individualities such as airway geometry, inhalation technique adherence, and breathing pattern. To understand how inhaled therapeutics behave in the lungs, we must apply boundary conditions more realistic than those used in pharmacopoeia methods. Current 3D prototyping methods or computational capacities allow us to imitate the *in vivo* situation using experimental or numerical simulation of the inhalation process in airway replicas with more realistic breathing conditions. Moreover, new computational tools are already being discussed as potential instruments for clinical areas to personalize healthcare.^40^

Several studies have already dealt with liposomal delivery.^3,24,41−45^ Nevertheless, Rudokas et al.,^9^ in their extensive review of liposomal delivery systems for the inhalation of anticancer therapeutics, emphasized the necessity of further research in the field of liposomal drug delivery, particularly in terms of formulation stability, nebulization mechanism, aerosol targeting, and minimization of aerosol deposition in the oropharyngeal region, since these are all crucial factors for inhalable liposomal therapeutics. Different nebulization technologies affect liposomal stability to different extents, and the resilience of the vesicles is both nebulizer-specific and formulation-specific.^46^ However, very few studies compared the effect of various nebulizer types on the stability of investigated liposomes.^6,17,48^ Hence, our research paper deals with all the mentioned aspects of liposomal systems development, focusing on the case of two selected liposomal systems.

Two recent studies^49,50^ investigated the influence of the inspiratory flow rate on particle sizes of the aerosol particles exiting the nebulizer. The influence was identified as well as a change in the particle size with the change in the flow rate. Moreover, this effect varied based on the device or the formulation.^49,50^ If the size of the generated particles changes significantly with the increasing flow rate, then it may affect lung deposition due to changes in *Stk* and inertial impaction. These findings encourage the investigation of this effect in the case of liposomal nebulization and nebulizer inhalation. The measured data can help to optimize the boundary conditions for future deposition predictions.

In this study, electrostatically and sterically stabilized liposomes were aerosolized using five commercially available nebulizers. The effect of this process was defined as a change in the physicochemical properties of liposomes. APSD was first assessed by an aerodynamic particle sizer (APS) for all of the selected nebulizers. The measurement of APSD was subsequently performed by an Andersen cascade impactor (ACI) as well, but only in the case of the nebulizer and liposomal formulation with the highest stability and FPF. The particle size changes during a full breathing cycle were investigated to assess the influence of the inhalation flow rate and cyclic breathing on the particle size distribution of aerosol emitted from the nebulizer. Ultimately, a fraction of the nebulized liposomal system deposited in a realistic upper airway replica during a steady and realistic normal breathing profile was evaluated.

## 2 Materials and Methods

First, the influence of nebulization performed by various nebulizers on the stability of liposomes with different compositions was investigated. Electrostatic stabilization of liposomes was achieved by phosphatidic acid (PA) and steric and electrostatic stabilization by polyethylene glycol bound to phosphatidylethanolamine (PEG).^24^ In both formulations, dipalmitoylphosphatidylcholine (DPPC) was chosen as the primary phospholipid because of its highest occurrence in pulmonary surfactants.^9,51,52^ Cholesterol (Chol) was chosen to reduce the leakage of the encapsulated drug during the nebulization process by increasing the membrane’s stiffness.^5^ According to Niven et al.,^53^ at least 30 mol % of cholesterol can decrease drug leakage from liposomes caused by their nebulization. The two types of liposomes that are used will be referred to in the following text as DPPC-PA-Chol and DPPC-PEG-Chol. Second, the APSD of the aerosols generated by the nebulization of these systems was measured by a TSI APS 3321 (TSI Inc., Minneapolis, MN). Five commercially available nebulizers were selected for nebulization of the liposomal solution: Pari LC Sprint (Air-jet nebulizer, PARI GmbH, Starnberg Germany), Pari LC Sprint Star (Air-jet nebulizer, PARI GmbH, Starnberg Germany), Aerogen Solo (Mesh nebulizer, Aerogen Ltd. Galway, Ireland), Pari eFlow Rapid (Mesh nebulizer. PARI GmbH, Starnberg, Germany) and Laica MD6026P (Ultrasonic nebulizer, Barbarano Mossano, Italy). APSD and the stability of liposomal systems nebulized by AJN were measured for two different nebulization flow rates to evaluate the influence of the flow rate on liposomal stability and particle size.

### 2.1 Materials

Phospholipids such as 1,2-dipalmitoyl-*sn*-glycero-3-phosphatidylcholine (DPPC, purity ≥ 99.0%), 1,2-dilauroyl-*sn*-glycero-3-phosphate (sodium salt, PA, purity ≥ 99.0%), and 1,2-dipalmitoyl-*sn*-glycero-3-phosphoethanolamine-*N*-[methoxy(polyethylene glycol)-5000] (ammonium salt, PEG5000-PE, purity ≥ 99.0%) were obtained from Avanti Polar lipids Inc., Alabaster, AL. Cholesterol (purity ≥ 99.0%) was purchased from Sigma-Aldrich Inc., St. Louis, MO. Fluorescently labeled phospholipid 1,2-dimyristoyl-*sn*-glycero-3-phosphatidylethanolamine by Atto 488 (Atto 488 DMPE, purity ≥ 80.0%) was also obtained from Sigma-Aldrich Inc., St. Louis, MO. The solvents for liposome preparation, chloroform, and methanol were obtained from Penta s.r.o. and Sigma-Aldrich Inc., St. Louis, MO, respectively. Pure water was obtained from ELGA LabWater Ltd., Lane End, UK.

### 2.2 Liposomal Preparation and Characterization

Liposomes were prepared by using a thin-film rehydration method, and their size was adjusted by using sonication. The phospholipids in the liposome formula (DPPC-PA-Chol or DPPC-PEG-Chol) were weighed and dissolved in chloroform and methanol (4:1, v/v). The amount of DPPC in liposomes was 0.7 mg/mL. The total concentration of cholesterol was 30 mol % because only this concentration could decrease the leakage of encapsulated drugs.^53^ The concentrations of phosphatidic acid and polyethylene glycol were 30 and 6 mol %, respectively, according to our previous results.^24^ See Table 1 for a better understanding of the composition of the investigated liposomes. Chloroform and methanol were evaporated, leaving a thin film of phospholipids, which was subsequently rehydrated by deionized water to create multilamellar vesicles. A probe sonication (model HD 3 200, Bandelin Electronic GmbH & Co. KG) was used to produce unilamellar vesicles in the case of DPPC-PA-Chol. A sonication water bath (model DT 31 H, Bandelin Electronic GmbH & Co. KG) was used for 15 min to resize vesicles composed of DPPC-PEG-Chol. PEG_5000_–PE cannot be incorporated into vesicles by probe sonication, so the ultrasonic bath was used.^6,24^ The size and zeta potential of the liposomes after preparation are summarized in Table 1.

**Table 1:** Composition of the Investigated Liposomes and Their Measured Size (*d*) and Zeta Potential (ζ)

	DPPC [mg/mL]	Chol [mol. %]	PA [mol. %]	PEG [6 mol. %]	*d* [nm]	ζ [mV]
DPPC-PA-chol	0.7	30	30		107 ± 3	–55 ± 4
DPPC-PEG-chol	0.7	30		6	111 ± 7	–7 ± 6

The fluorescence labeling of liposomes was performed by adding Atto 488 DMPE to the vesicle composition before the formulation of liposomes. The total concentration of Atto 488 DMPE after a 5-fold sample dilution was 0.01 mg/mL. The linear calibration curve of Atto 488 was performed for the range of concentration 10^–9^–10^–4^ mg/mL and measured with a spectrofluorimeter FS5 (Edinburg Instruments Ltd., UK) to establish the particle mass deposited in the individual segments of the Andersen cascade impactor (see section 2.3.2) and upper airways realistic replica (see section 2.5.2).

Liposome size distribution was determined by a ZetaSizer Nano ZS instrument (Malvern Instruments Ltd.). The sample was illuminated by a He–Ne laser, and the scattered light was collected at an angle of 173°. The diffusion coefficient *D*_C_ of the measured sample was obtained from the autocorrelation curve. The hydrodynamic diameter *d*_H_ was calculated by the Stokes–Einstein equation *d*_H_ = *k*_B_*T*/3π*ηD*_C_ where *k*_B_ is the Boltzmann constant, *T* is the temperature, and η is the solvent viscosity. The *d*_H_ in all results corresponds to the value *Z*_Ave_ (intensity-weighted mean hydrodynamic size) gained by the cumulant analysis. All measurements were carried out at a constant temperature (25 °C) after 60 s of calibration. Mean values and standard deviations of size were calculated from three independent measurements.

The investigated liposomal systems were nebulized by all of the nebulizers, and aerosol particles were subsequently collected. In the case of the mesh nebulizers, the outlet was connected directly to the vial neck and sealed. The collection setup described in refs (6) and (52) was used for air-jet nebulizers. A dedicated Pari Boy Pro air compressor powered the air-jet nebulizers and operated with a nebulization flow rate of 4.5 L/min. The total fraction of vesicles resistant to the nebulization process was assessed by the vesicle size distributions. The vesicle size distribution measured before nebulization was subtracted from the vesicle size distribution after the nebulization. This difference evaluated the changes in the measured intensity in each class of vesicle size. During nebulization, vesicles can be disintegrated by mechanical stress or other effects, but they can also be reintegrated into a vesicle of larger diameter.^24^ Therefore, to determine the extent of vesicle damage, only the negative change in intensities was included in the total vesicle damage since including both the increase and the decrease of vesicles would overestimate the value of the total vesicle damage (the damage evaluation method is used and described in more detail in ref (6)). The fraction of resistant vesicles was then evaluated as FRV = 100 – FDV [%], where FRV is the fraction of resistant vesicles, and FDV is the fraction of damaged vesicles.

### 2.3 Aerosol Particle Size Distribution

Aerosol particle size distribution (APSD) was first measured by an aerodynamic particle sizer (APS, TSI Inc., Minneapolis, MN), which is faster and more convenient than the standard impactor method. Based on these results and the liposomal stability data, the most promising combination of a nebulizer and liposomal system was selected and then also measured by the Andersen cascade impactor (ACI).

#### 2.3.1 Aerodynamic Particle Sizer Measurement

**Figure 2:**
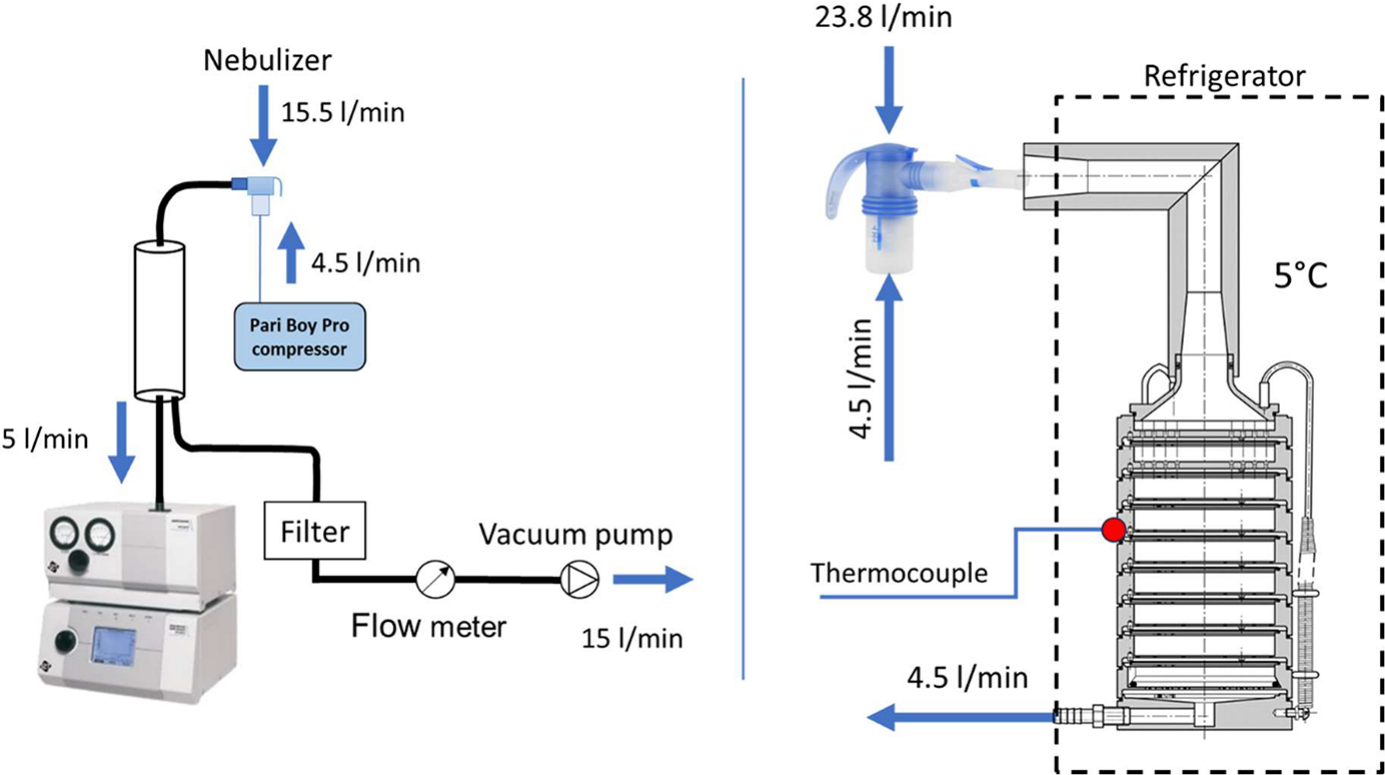
APSD measuremet scheme (APS measurement:left; ACI measurement: right).

The measurement setup was similar to the study of Wang et al.^54^ The scheme of the measurement is shown in Figure 2. Aerosol was nebulized into a chamber from where it was sampled into the APS (Figure 2) at a flow rate of 5 L/min. The second bypass branch led to the vacuum pump from the chamber, and the bypass flow rate was 15 L/min. The total inhalation flow rate sucked out through the nebulizer was hence 20 L/min. The APSD of ambient air was measured before the sampling and subtracted from the measured aerosol samples during the analysis. Besides the investigated liposomal systems, the normal saline solution (0.9% NaCl) APSD was measured to see the effect of the liposomes on the aerosol particle size. Five samples were measured for each case, and mass median aerodynamic diameter (MMAD), geometric standard deviation (*GSD*), and FPF were evaluated. FPF was considered as the fraction of the aerosol mass in particles smaller than 5 μm.

#### 2.3.2 Andersen Cascade Impactor Measurement

An eight-stage ACI was used to investigate the APSD of the selected nebulizer–liposomal system combination further. It was placed in the refrigerator and cooled to the temperature of 5 °C to eliminate the effect of particle evaporation on their path through the device (according to the recommendation in the *European Pharmacopoeia*). The outer surface temperature was monitored at the bend of the induction port and the third ACI stage (Figure 2).

The ACI operated in the flow rate configuration of 28.3 L/min, the lowest possible flow rate at which it can be used. Before the measurement, a tightness test was performed, and the flow rate through the ACI was monitored by a Mass Flow Meter 4040 (TSI Inc., Minneapolis, MN). The nebulizer was placed at the inlet of ACI using a modeled adapter.

The liposomal system had to be labeled to allow evaluation of the particle mass deposited in the individual segments of ACI (see section 2.2), so the phospholipid fluorescently labeled with ATTO 488 was incorporated into the vesicle. APS measured the effect of the dye on the APSD, and no influence on the labeling was detected.

The aerosol was inhaled into the ACI system for 1 min. After exposure, the particles deposited on the inner surface of the instrument were extracted with distilled water, and the amount of dye in each ACI segment was analyzed with a spectrofluorimeter FS5 (Edinburg Instruments Ltd., UK). Subsequently, MMAD, *GSD*, and FPF were calculated from the gained values according to the approach described in ref (55). The scheme of the APSD measurements is shown in Figure 2.

### 2.4 Effect of Flow Rate and Breathing Pattern on the Particle Size

The changes in the aerosol particle size during the cyclic breathing regime and various inspiration flow rates were measured by Phase Doppler Anemometry. The particle size distribution was first measured at steady flow rates of 8, 12, 20, 40, and 60 L/min. Subsequently, the cyclic breathing profile was used to measure this effect. The simulator has five pistons with programmable movement, allowing it to simulate any breathing profile. The pneumatic valves in the simulator allow the expiration and inspiration to be performed through the same or different paths of the measuring assembly, so it can perform the inhalation through the model/inhaler and exhalation to the ambient air (bypassed). Two different cycles were performed. The first cycle had a tidal volume of 0.5 L, and the period was 3 s. The second cycle had a tidal volume of 1.5 L, and the period was also 3 s, which approximately corresponds to the data reported in ref (56) for sitting awake adults and adults during heavy exercise. This investigation was performed only for the Pari LC Sprint Star nebulizer.

**Figure 3:**
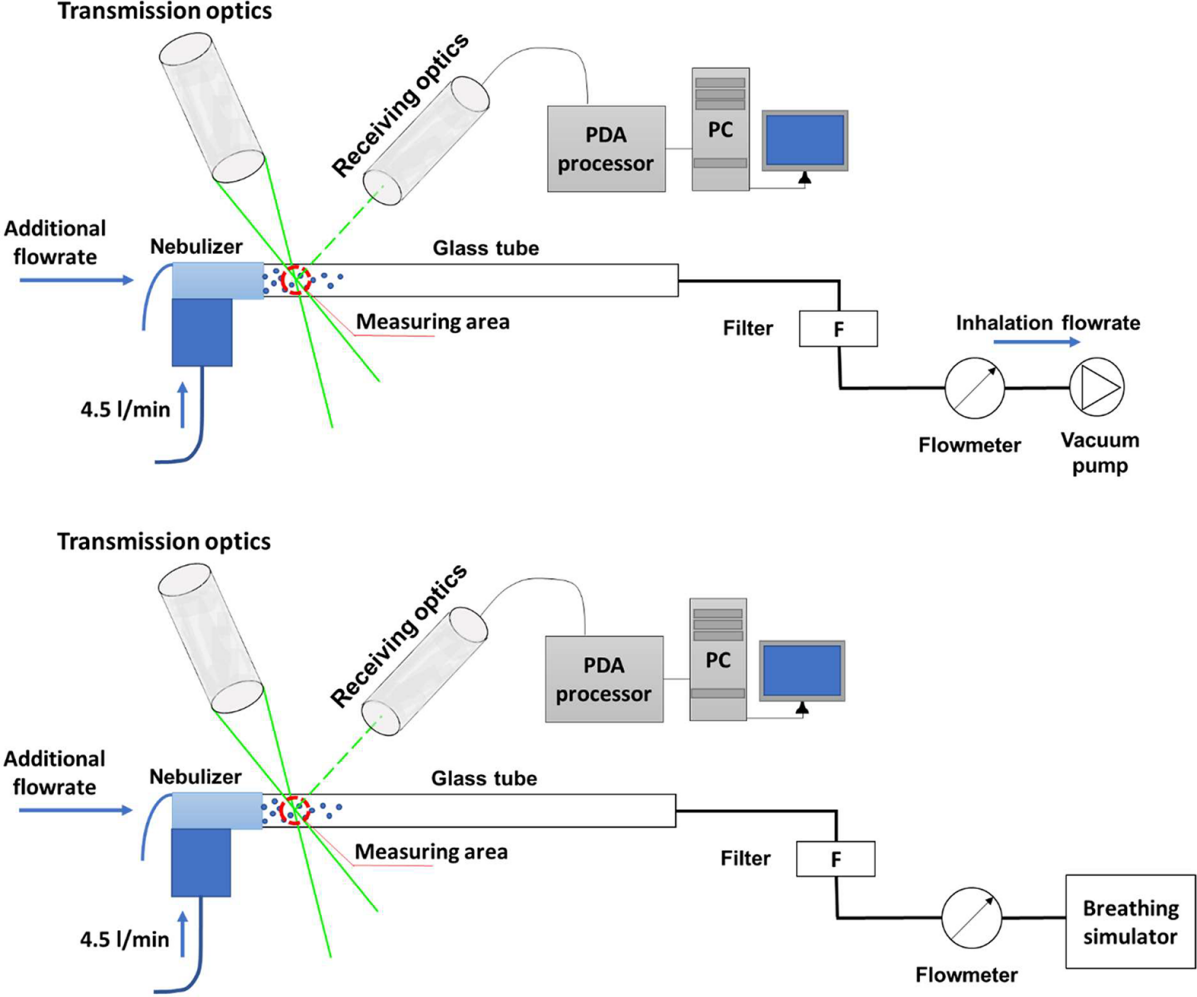
Scheme of the PDA measurement. Upper: steady flow rate measurement; Lower: tidal breathing measurement.

Particle sizes were determined using a 1D fiber-based PDA measurement system (Dantec Dynamics A/S Skovlunde, Denmark). A laser beam with a power of 300 mW was emitted from a 2017 Spectra Stability 2017 Ar-ion Laser (Spectra-physics, USA). It was separated into individual wavelengths in a 60 × 41 Transmitter box. Only the wavelength of 514.5 nm was used for the measurement. The transmitter box split the beam into two and shifted one beam by about 40 MHz to allow for a zero velocity measurement. The beams were focused onto an optical fiber leading to the transmitting optics 60 × 81, equipped with a 1.98× beam expander and 310 mm lens. The lens focused the beams and formed a measuring volume with dimensions of 0.076 mm × 0.076 mm × 0.59 mm. The first-order refracted light from particles was captured by receiving optics (57 × 50) equipped with a 310 mm focal length lens and 0.1 mm slit to reduce measuring volume length. The scattering angle was 70°, and the maximum measured particle size was 39 μm. Either 20,000 samples or a 15 s acquisition duration was achieved, depending on whichever occurred first. The Dantec BSA software 5.2 was used to control the measurement. The nebulizer outlet was connected to a glass tube with an inner diameter of 16 mm and a length of 20 cm by a 2 cm long piece of hose. The output of the glass tube was connected to a filter and a Mass Flow Meter 4040 (TSI Inc., Minneapolis, MN). A three-way control valve was placed behind the flowmeter and connected to a vacuum pump/breathing simulator. The laser cross section (measuring volume) was placed directly behind the nebulizer connection, 1.5 cm from the output. The glass tube was placed horizontally. The measuring volume was located in the middle of the tube height, and the particle size data were recorded at seven positions on the horizontal axis of the tube cross-section. The center of the coordinate system was placed in the middle of the tube cross-section so that the horizontal axis corresponded to the *X*-axis of the coordinate system. Measured positions were for the *X*-axis: −6 mm, −4 mm, −2 mm, 0 mm, 2 mm, 4 mm, and 6 mm; for the *Y*- and *Z*-axes, the coordinates were 0 mm. The PDA was mounted to a 3D positioning system Isel (Eichenzell, Germany) with an accuracy of ±0.05 mm and alignment accuracy relative to the tube centerline of roughly ±0.5 mm. The scheme of PDA measurement is shown in Figure 3.

The effect of the steady flow rate was assessed as a change in the *D*_*v*50_ parameter of emitted aerosol with various flow rates. Particle diameter was measured in all of the mentioned positions, and *D*_*v*50_ was evaluated as a weighted average of *D*_*v*50_ in different positions. Such weighted *D*_*v*50_ average value is the so-called Integral Diameter (*ID*) and is calculated according to the equation:
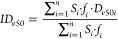
where *S*_*i*_ is the area of the annular zone of the tube cross section bounded by a 1 mm distance in the inner direction and 1 mm distance in the outer direction from the measuring position is the data rate in the measuring point, and *D*_*v*50_ is the median of the volumetric particle size distribution.

Particle size data recorded during the breathing cycle were divided into inhalation and exhalation. The inhalation part was then divided into five equal time sections, and particle size distribution (*PSD*) parameters were evaluated separately for each time section separately. *D*_*v*50_ and *GSD* were evaluated for each position, and the integral diameter was calculated in the same way as in the case of a steady flow regime. The flow rate of the compressed air passing through the nebulizer (4.5 L/min) and the flow rate sucked by the vacuum pump was monitored during the measurement by two mass flow meters Mass Flow Meter 4040 (TSI Inc., Minneapolis, MN). It is necessary to emphasize that the PDA method provides the particle size data in terms of optical diameters. Such data differ from aerodynamic particle size measured by APS or ACI due to different physical principles used for measurement.

### 2.5 Aerosol Deposition in the Upper Airways

**Figure 4:**
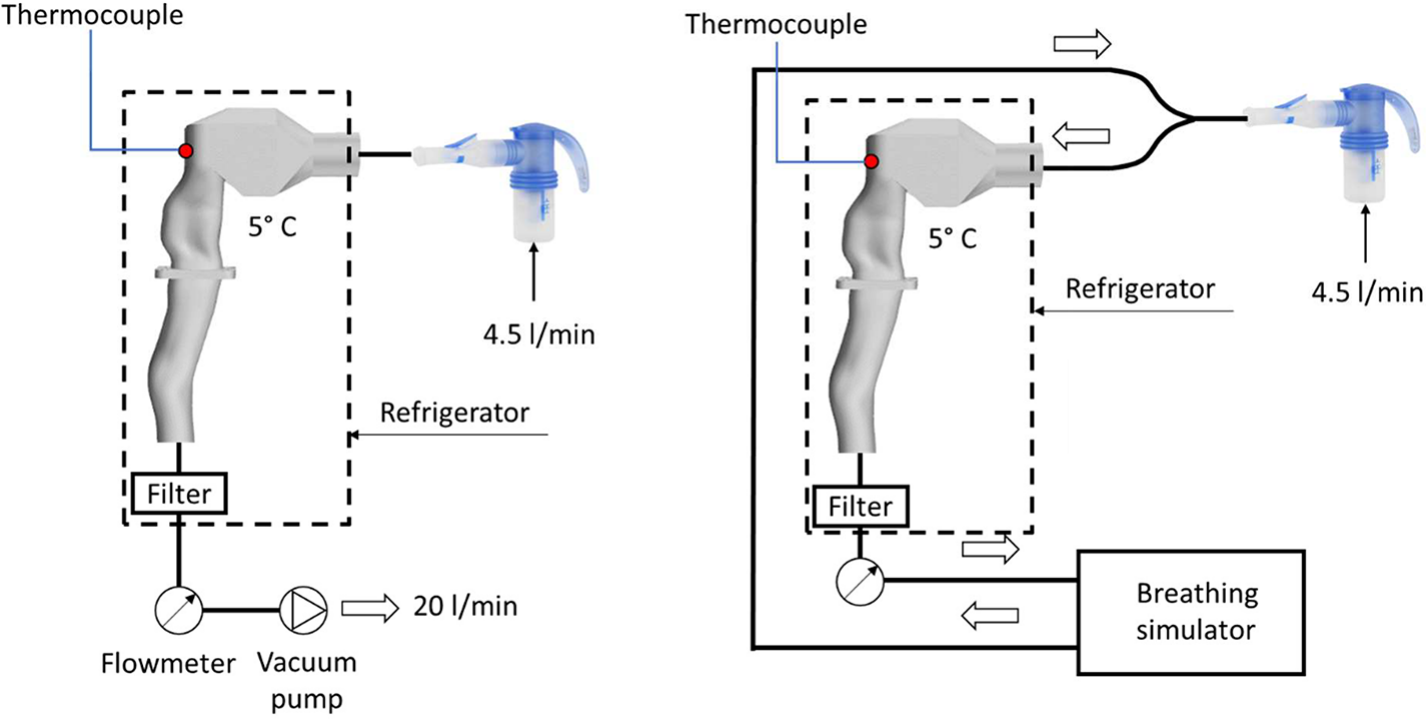
Scheme of the deposition measurements.

The realistic replica of the upper airways consisted of the mouth cavity, pharynx, and larynx. The replica was created from the cast of an adult male mouth and upper airways (dental impression, cast from a cadaver), and the preparation of such a replica is described in refs (57) and (58). Before measurement, the connection of these segments was sealed by sanitary silicone. Liposomes were labeled with an ATTO 488 fluorescence probe (see section 2.2) in the same way as in the case of ACI measurement. The scheme is shown in Figure 4.

#### 2.5.1 Measurement of Aerosol Deposition during Steady Flow Regime

An MF-Millipore membrane filter with a diameter of 47 mm and a pore size of 0.8 μm was placed downstream of the trachea. As in the case of ACI measurements, the replica was cooled to a temperature of 5 °C to eliminate the effect of droplet evaporation, and the temperature was monitored on the outer surface of the mouth cavity and trachea. Mass Flow Meter 4040 (TSI Inc., Minneapolis, MN) was used to monitor the flow rate. The scheme is shown in Figure 4.

Aerosol was inhaled into the replica for 1 min with a flow rate of 20 L/min, the average inhalation flow rate calculated from the values for normal tidal breathing published in.^59^ Afterward, the replica was disassembled, and the deposited aerosol was extracted from the inner surface of the segment and the membrane filter with distilled water in an ultrasonic cleaner. The amount of dye was subsequently assessed using an FS5 spectrofluorimeter (Edinburg Instruments Ltd., UK). From the measured data, the deposition fraction in the nebulizer mouthpiece, the upper airway segment, the trachea, and the filter below the trachea were evaluated.

#### 2.5.2 Realistic Inhalation Measurement

A breathing simulator^60^ was used for the flow conditions setting. The membrane filter placed below the trachea was connected to the inspiration path of the breathing simulator. The nebulizer mouthpiece containing the exhalation valve was connected to the Y-piece hose branching. One way (the inspiration path) of this branching continued to the mouth cavity. The second way was connected to the expiration path of the breathing simulator. It allowed for an assessment of aerosol deposition within the mouthpiece during the breathing profile and ensured a realistic breathing pattern through the nebulizer. The particles would have accumulated more inside the mouthpiece if the expiration had not been routed through the nebulizer. They could have coagulated during the expiration period, artificially increasing the deposition fraction in the mouthpiece or upper airways. By this assembly, we ensured a realistic situation inside the nebulizer.

**Figure 5:**
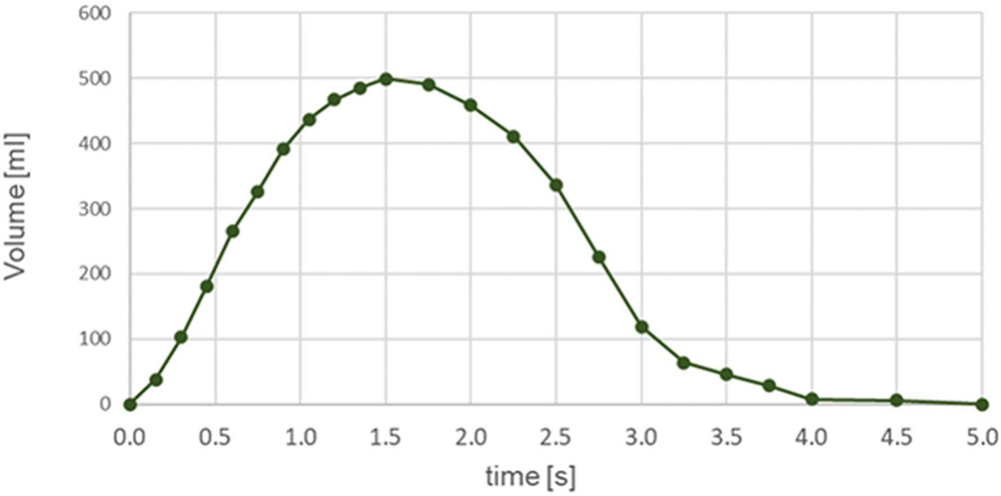
Breathing profile used for the realistic breathing deposition measurement.

The realistic breathing profile was prepared from the respiratory records of 17 healthy subjects and is described in ref (59). The tidal volume was 500 mL, and the cycle consisted of inspiration (1.5 s), expiration (2.5 s), and breath-hold (1 s). The volume breathing profile is shown in Figure 5. The peak inspiration flow rate was 33.64 L/min and occurred at 0.525 s after the beginning of inhalation.

For this **in vitro** upper airway deposition experiment, the liposomes with phospholipid fluorescently labeled with ATTO 488 (section 2.2) were used, as in the case of steady regime deposition or ACI measurement. The nebulizer Pari LC Sprint Star was used for this investigation and operated with a nebulization flow rate of 4.5 L/min.

During exposure, 100 breathing cycles were performed. After exposure, the deposited aerosol was extracted from the surfaces of the nebulizer mouthpiece, the connecting hoses, the upper airway replica, the trachea replica, and the filter using an ultrasonic bath. The samples thus prepared were analyzed with a FS5 spectrofluorimeter (Edinburg Instruments Ltd., UK), and the mass of the dye in the samples was evaluated. Subsequently, the deposition distribution within the replica was calculated from these values.

## 3 Results and Discussion

### 3.1 Stability of the Liposomal Systems against the Nebulization Process

**Figure 6:**
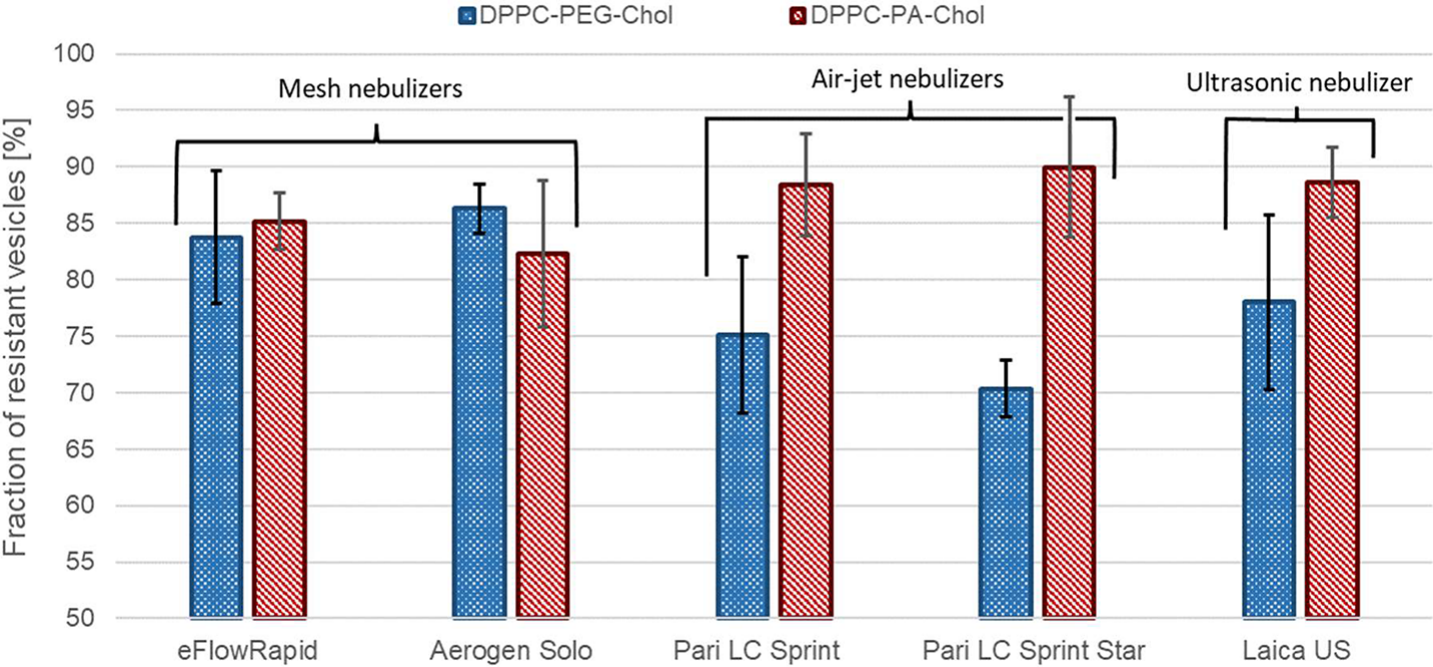
Total change in vesicle size distribution after the nebulization by various nebulizers for DPPC-PA-Chol and DPPC-PEG-Chol.

The influence of different types of nebulization technology on the vesicle size distribution of the chosen liposomal composition was evaluated as the total change of the vesicle size distribution (see section 2.2), which is the same method as described in ref (6), and these results are summed up in Figure 6. The error bars indicate the standard deviations calculated from three measurements. The whole vesicle size distributions are displayed in Supporting Information. A marked difference in the stability of liposomes during nebulization was identified between the investigated liposomal systems, especially in the case of AJNs. The electrostatically stabilized vesicles (DPPC-PA-Chol) showed significantly higher resistance against the nebulization process in comparison to the sterically and electrostatically stabilized liposomes (DPPC-PEG-Chol) for AJNs. In the case of vibrating mesh nebulizers (Aerogen Solo and eFlow Rapid), the viability during nebulization was similar for both liposomal systems. Liposomes composed of DPPC-PA-Chol did not alter their physicochemical properties after ultrasonic nebulization either. These results show that DPPC-PA-Chol liposomes prove to have high stability against different nebulization processes and could be a suitable drug delivery system for pulmonary drug delivery. The important finding supported by the results in Figure 6 is that different liposomal systems may exhibit varying behaviors during nebulization by the same nebulizer. Their resistance against the damage caused by nebulization can significantly differ, even when using the same nebulizer. This finding implies that generalizing the optimal nebulizer for liposomal systems is an unsuitable and limited approach. Therefore, it is essential to consider the specific characteristics and behavior of each liposomal system during nebulization, which can differ significantly from one system to another.

### 3.2 Aerodynamic Particle Size Distribution

#### 3.2.1 Aerodynamic Particle Size Measurement

The APSD of all liposomal systems nebulized by all investigated nebulizers was measured with an APS device. MMAD, *GSD*, and FPF were evaluated from the measured APS data and are shown in Table 2. The method for calculation of these parameters is described in ref (55). According to the particle size results, the composition of the liposomal systems did not significantly influence the APSD parameters. However, a significant difference was found compared with the normal saline solution (Table 2). The current study used the APS measurement to compare available nebulizers. The APS results were used to select a suitable nebulizer for further investigation. The critical parameters for the selection were the resistance of the liposomes against the nebulization process and the largest FPF. According to these criteria, the most suitable combination is Pari LC Sprint Star with the DPPC-PA-Chol (FPF = 51.61 ± 1.67, fraction of resistant vesicles of 89.97 ± 6.18%). Relatively high FPF and resistance were also observed in the case of the Laica ultrasonic nebulizer. However, since ultrasonic nebulizers are unsuitable for thermolabile ingredients and the scope of applications is limited, the Laica nebulizer was not selected for the next steps.

**Table 2:** Parameters of the Aerosol Aerodynamic Particle Size Distribution Measured by APS

	MMAD [μm]	*GSD* [—]	FPF [%]
***Normal Saline***			
Aerogen	5.54 ± 0.22	1.89 ± 0.05	42.85 ± 3.31
eFlowRapid	4.78 ± 0.44	1.97 ± 0.18	51.40 ± 4.16
Pari LC Sprint	5.72 ± 0.13	1.80 ± 0.02	36.07 ± 1.83
Pari LC Sprint Star	3.27 ± 0.60	2.78 ± 0.71	67.14 ± 2.82
Laica	4.63 ± 0.19	1.77 ± 0.11	56.66 ± 3.90
***DPPC-PEG-Chol***			
Aerogen	7.36 ± 0.15	1.48 ± 0.02	24.75 ± 1.10
eFlowRapid	5.56 ± 0.24	1.66 ± 0.05	37.82 ± 3.98
Pari LC Sprint	6.17 ± 0.10	1.68 ± 0.02	26.44 ± 0.87
Pari LC Sprint Star	5.03 ± 0.08	1.42 ± 0.04	49.07 ± 2.21
Laica	5.19 ± 0.07	1.65 ± 0.05	45.30 ± 1.93
***DPPC-PA-Chol***			
Aerogen	6.89 ± 0.19	1.60 ± 0.03	15.94 ± 0.81
eFlowRapid	5.33 ± 0.14	1.67 ± 0.06	41.03 ± 3.72
Pari LC Sprint	6.21 ± 0.31	1.67 ± 0.02	26.23 ± 2.51
Pari LC Sprint Star	4.94 ± 0.06	1.40 ± 0.02	51.61 ± 1.67
Laica	5.09 ± 0.09	1.65 ± 0.04	47.72 ± 2.30

#### 3.2.2 Andersen Cascade Impactor Measurement

**Figure 7:**
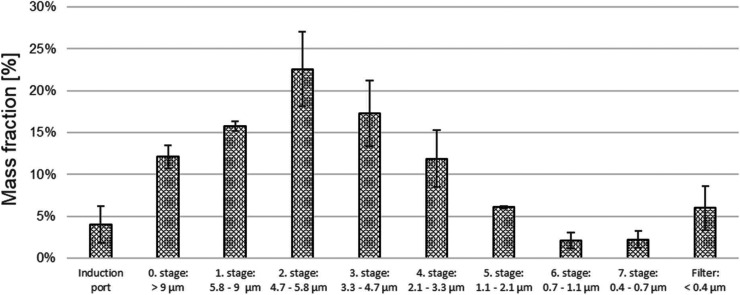
Mass fraction deposited on segments of ACI for DPPC-PA-Chol and the Pari LC Sprint Star nebulizer.

The DPPC-PA-Chol liposomal system was fluorescently labeled with ATTO 488, nebulized by Pari LC Sprint Star, and measured by the ACI. From the measured mass of ATTO 488 dye in the impactor segments, the MMAD, *GSD*, and FPF were calculated using the method described in ref (55). Since the labeling dye was incorporated directly into the liposomal vesicles, the measured APSD corresponds directly to the number of the vesicles, not only to the solution deposited mass. MMAD of the dyed liposomal formulation DPPC-PA-Chol was 4.89 ± 0.19 μm, *GSD* was 1.85 ± 0.10, and FPF was 51.70 ± 3.50%. This result is in very good agreement with the APS data. The mass fraction in each part of ACI is plotted in Figure 7. The error bars represent the standard deviations calculated from three measurements.

### 3.3 Measurement of Particle Size Changes Induced by Different Inhalation Flow Rate and Breathing Cycle

**Figure 8:**
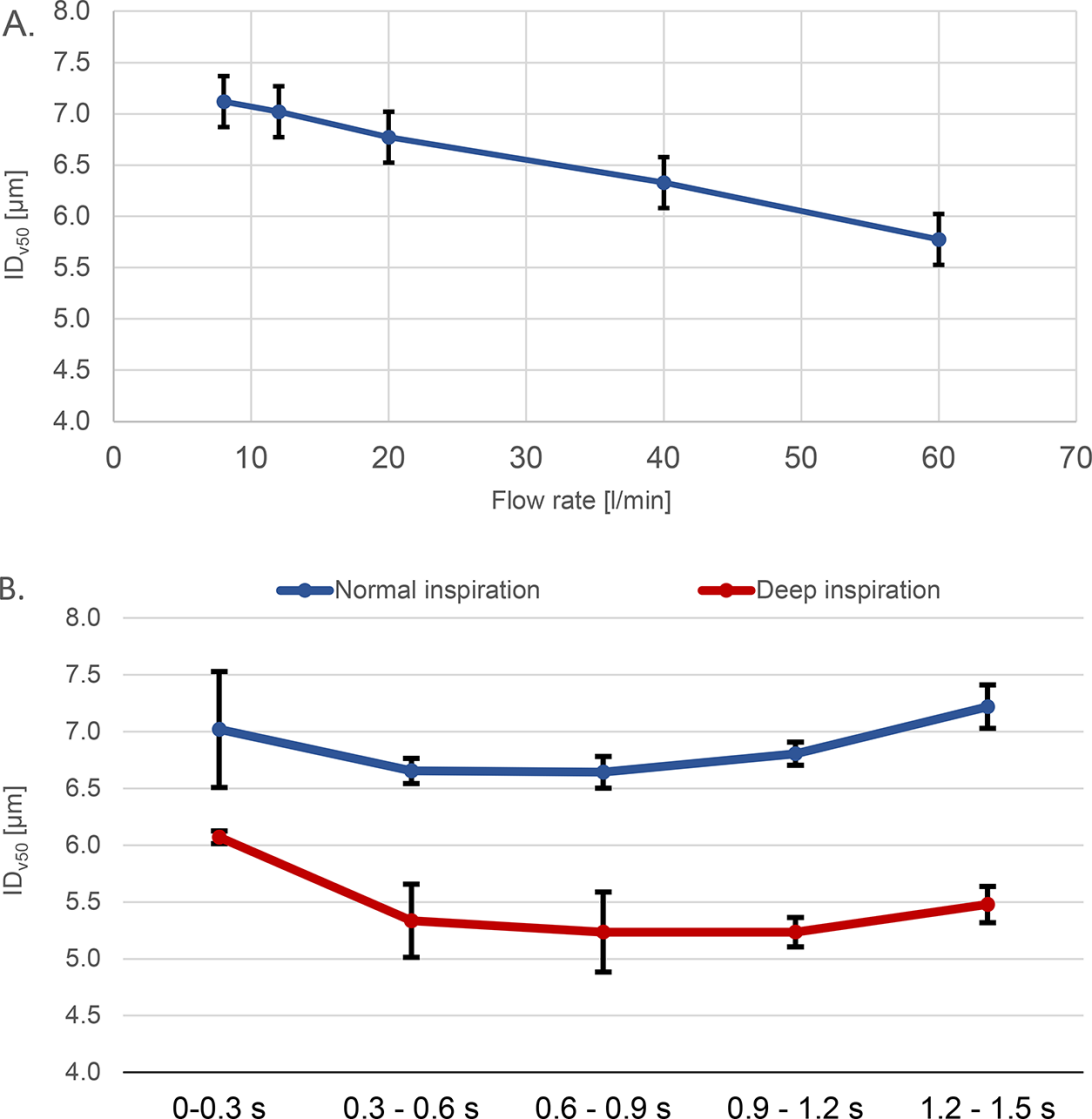
A. Change of the volumetric particle size distribution median with increasing flow rate. B. Change of the volumetric particle size distribution median during the breathing profile.

The PDA measurement of the particle size distribution of the aerosol generated by the Pari LC Sprint Star nebulizer shows a slight dependence on the inspiration flow rate. It can be seen in Figure 8A that the median of volumetric particle size distribution decreased by 1.34 μm with the flow rate change from 8 to 60 L/min. The change of particle size with the flow rate is linear. The error bars were determined as a standard deviation of from five measurements of the steady regime and four breathing cycle measurements of the cyclic regime. The development of the aerosol particle size during normal and deep inspiration is shown in Figure 8B. An influence of the flow rate during the breathing cycle can be seen in the case of normal tidal breathing, and an even more significant effect was detected during the deep breathing cycle, where the values of in the middle of the inspiration are smaller by approximately 1.40 μm than the values of normal tidal breathing. The reason for this behavior could be 2-fold: First, the higher inspiration flow rate causes more intensive mixing of the air from the nebulizer with the dry ambient air, which can lead to droplet evaporation; Second, the high concentration of particles inside the nebulizer chamber in the case of a lower flow rate leads to particle coagulation. The *ID*_*v*50_ varies between 7.22 and 6.64 μm during normal tidal breathing and between 6.06 and 5.23 μm during deep breathing, resulting in a relatively small difference of 0.58 and 0.84, respectively. This size change will probably not be significant from the perspective of pulmonary drug delivery.

### 3.4 Deposition of the DPPC-PA-Chol Liposomal System within the Upper Airways

**Figure 9:**
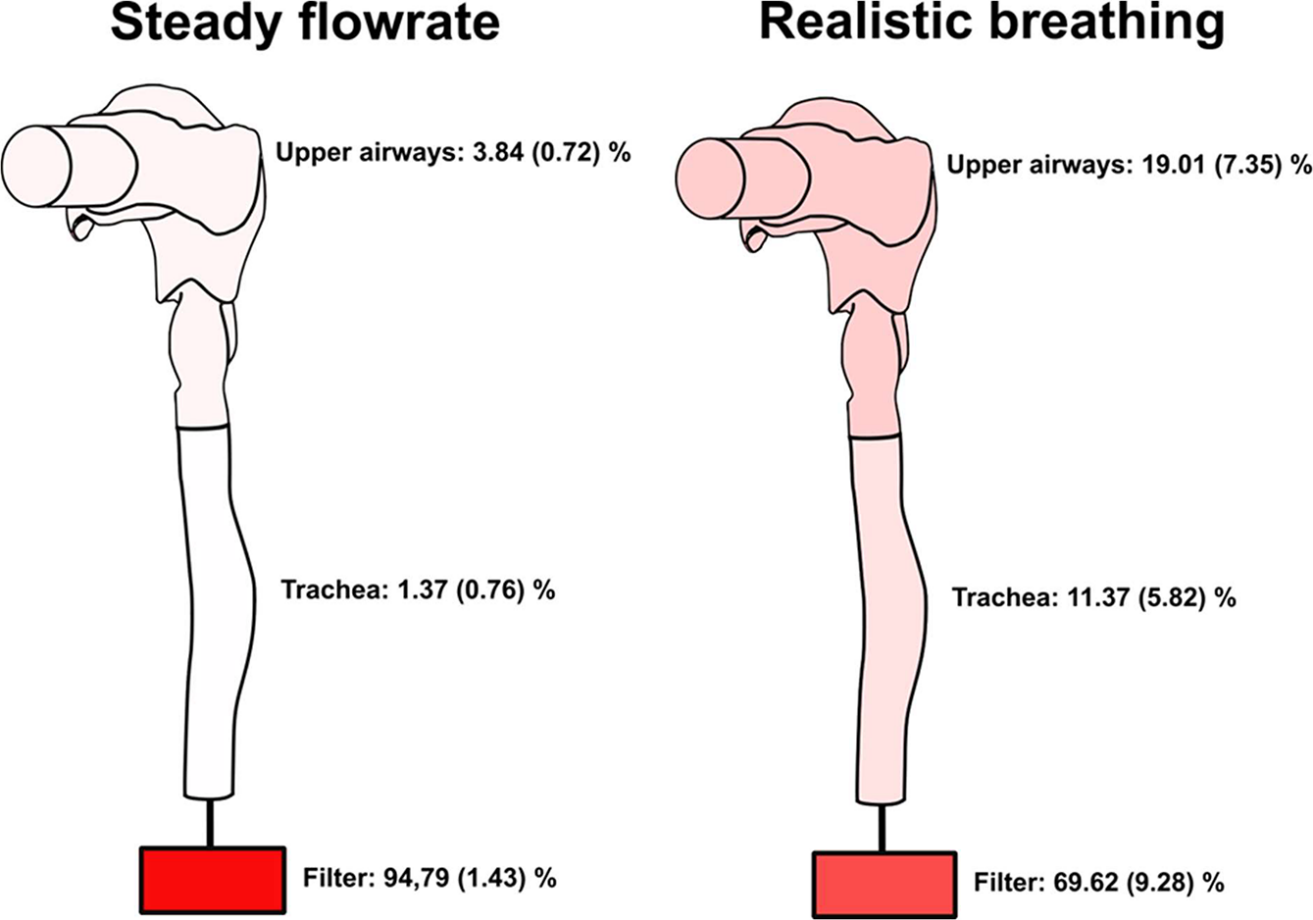
Deposition of DPPC-PA-Chol in upper airways replica during steady flow rate and realistic inspiration (standard deviations from three experiments are shown in the brackets).

The distribution of deposition fractions within the extrathoracic (ET) airways, the trachea, and the filter connected below the trachea is shown in Figure 9. The results show that significantly fewer particles penetrated the upper respiratory airways and trachea during the realistic breathing profile. Approximately 69,62 and 94,79% of the total aerosol mass deposited on the outlet filter during realistic normal breathing and steady flow regime, respectively. The results also suggest that in the case of nebulized aerosol with MMAD of 4.89 ± 0.19 μm, *GSD* of 1.85 ± 0.10 μm, and FPF of 51.70 ± 3.50% (measured by ACI), around 70% of aerosol which enters the airways can penetrate below the trachea of the model during the realistic breathing pattern. As was described in the Introduction, the FPF concept was created for dry powder inhalers and pressurized metered dose inhalers, which require fast and deep inhalation. However, in that case, it overestimated the entire lung deposition. In our data, the FPF concept seems to underestimate lung deposition.

Moreover, exhalation was not considered in this study, and all of the particles that penetrated deeper downstream of the trachea were collected on the filter. However, in the case of actual usage, some of these particles may be exhaled, so they may be inefficient for treatment. It would decrease the fraction deposited in lower regions compared to our measurement. In the case of steady flow measurement, it is interesting that the fraction deposited at the induction port of ACI and in the ET segment of the airway replica was nearly identical: 4.03 ± 2.19% deposited at the ACI induction port (28.3 L/min, steady) and 3.84 ± 0.72% deposited in the ET segment of the replica (20 L/min, steady). The difference in the deposition data is expected because of the difference in the operating flow rate. Although the ACI is not a device to imitate lung deposition and is dedicated to particle size measurement, the induction port was designed to imitate mouth–throat deposition.^61−63^ According to our results, this analogy is applicable only in the case of a steady flow rate as the deposition in the ET region during realistic breathing was significantly higher, approximately 19%. This difference underlines the necessity of more realistic deposition measurements for inhaler testing than cascade impactor studies to create an image of the aerosol behavior in the lungs, which can also be valuable for clinicians in planning the treatment.

An *in vitro* deposition study^64^ investigated aerosol distribution inside a lung model with the same ET airways replica used in the study presented here. The stationary flow rates of 15 and 60 L/min and particles of 4.3 μm were used. The ET deposition fraction acquired in the mentioned study for 15 and 60 L/min was approximately 2.5% and 6.5%, respectively. These results agree reasonably well with the results of this study. Moreover, obtained deposition data are also supported by the results of Heyder et al.,^65^ who employed monodispersed aerosol. They calculated the deposition fractions for selected airway regions based on *in vivo* data. The upper airway deposition fraction of particles with a diameter of 5 μm for a 4 s long breathing profile with a tidal volume of 0.5 L (a very similar breathing pattern as the one used in this study) was 24%.^65^ In the case of the deposition experiment presented here, the fraction of aerosol deposited in the segment of upper airways was approximately 20%, with the MMAD of 4.89 μm (*GSD* = 1.85) measured by ACI for this aerosol. The deposition results strongly depend on the individual airway geometry, breathing pattern, etc. Our replica represents the geometry of only one specific patient. From the previous *in vivo* studies,^66^ we can see significant differences in airway deposition between the individuals, so it is complicated to generally assume the efficacy of drug delivery to the lungs from measured data. However, since the conditions of our measurements mimic a realistic situation, the results have a higher informative value than standard testing methods. Despite this fact, the measured deposition data bring the approximate prediction of upper airway deposition of the DPPC-PA-Chol liposomal system nebulized by the Pari LC Sprint Star nebulizer, and this shows how strongly it can differ from the predictions according to the FPF concept or cascade impactor measurements. This also confirms the importance of the role of a realistic breathing profile and a correct inhalation technique for airway deposition.

From the data mentioned above, it can be assumed that the DPPC-PA-Chol is a promising carrier system with high resistance against damage during nebulization (approximately 90%) when administered by air-jet nebulizer Pari LC Sprint Star (approximately 70% of liposomes were delivered below the trachea).

## 4 Conclusion

The resistance of the DPPC-PA-Chol and DPPC-PEG-Chol liposomal systems against nebulization by various nebulizer technologies was quantified. The liposomal system DPPC-PA-Chol showed relatively high resilience, and its fraction of intact vesicles varies between 80–90% for nebulization by all investigated nebulizer technologies. The resilience of the DPPC-PEG-Chol system was high only in the case of vibrating mesh nebulizers. However, during the air jet and ultrasonic nebulization, the damage to the vesicles was significantly more intensive. The highest fraction of intact vesicles was observed in the case of Pari LC Sprint Star and Laica US nebulizers. Since ultrasonic nebulization can be an obstacle for some active pharmaceutical ingredients, the air-jet nebulization (Pari LC Sprint Star) of the DPPC-PA-Chol liposomal carrier can be considered the most suitable choice from the investigated variants.

Aerodynamic particle size distribution data measured by the Aerodynamic Particle Sizer showed the largest FPF in the Pari LC Sprint Star nebulizer case. The DPPC-PA-Chol system’s stability and FPF during its nebulization by the Pari LC Sprint Star nebulizer were the reasons for selecting this system and this nebulizer for further measurements. Aerodynamic particle size measurement of the DPPC-PA-Chol system nebulized by Pari LC Sprint Star was also measured by an Andersen cascade impactor. MMAD measured by the cascade impactor is 4.89 ± 0.19 μm and *GSD* = 1.85 ± 0.10 μm. This result is in good agreement with the APS data.

The development of particle size distribution during breathing through the nebulizer and its change with the increasing flow rate was measured by PDA. Particle size was inversely proportional to the flow rate, and the increase from 8 to 60 L/min caused a decrease in *D*_*v*50_ by 1.34 μm. The size variation was even more minor during the unsteady regime. The particle size varied from 7.22 to 6.64 μm (the span of 0.58 μm) and from 6.07 to 5.24 μm (the span of 0.84 μm) during normal tidal and deep breathing, respectively. The regional deposition of the inhaled aerosol depends not only on the size but also on the lung geometry. The breathing profile also plays a significant role.^66^ Moreover, the breathing patterns and airway geometry are the parameters with high intrasubject variability. From this point of view, the size change of 0.58 μm during normal breathing seems relatively insignificant with regard to drug delivery.

The *in vitro* deposition measurement of the liposomal DPPC-PA-Chol system was performed on a realistic upper airway replica during a steady regime (20 L/min) and a realistic breathing profile. In the case of a steady flow regime, more than 90% of the aerosol mass that entered the airway model could penetrate below the trachea. In the case of the realistic breathing cycle, this fraction decreased to approximately 70%. It is essential to emphasize the difference between drug delivery during stationary and realistic inhalation. Since cascade impactors conventionally perform the aerodynamic assessment of the inhalation products with a steady flow rate, the nonrealistic flow rate conditions might overestimate the lung deposition prediction. Therefore, it cannot tell us much about the regional lung aerosol deposition.

Moreover, according to the ACI measurement, the FPF was only approximately 50%, but the *in vitro* deposition results showed a much higher deposition fraction below the trachea. This indicates the incorrectness of the assumption that FPF is a direct predictor of the effectiveness of drug delivery to the lung.^66^ The significant difference between the FPF and deposition fraction below the trachea is also related to the specific airway geometry.^67−69^ Although the regional aerosol lung deposition is strongly patient-specific, the results acquired on realistic replicas have a higher informative value about drug delivery than the standard cascade impactor method or fine particle fraction concept. According to our data, in both cases (steady flow and realistic inhalation), the ability of the aerosol to penetrate below the upper airways was very high.

The stability results show that it is inappropriate to generally select one suitable nebulizer for all of the liposomal systems since high differences in the resistance of various liposomes to the same nebulizer were observed. On the contrary, it is necessary to fit the nebulization technology specifically for the selected liposomal system. For example, the highest stability was observed in the case of the DPPC-PA-Chol system nebulized by Pari LC Sprint and Pari LC Sprint Star nebulizers, but the resilience of DPPC-PEG-Chol against nebulization by these two nebulizers was the poorest among the tested cases. The stability of the formulation is not the only criterion in the selection of the nebulizer technology; the aerodynamic parameters of the aerosol and its deposition in respiratory traction also play an essential role. This study described the potential of a selected liposomal system for pulmonary drug delivery to the lung from both of these aspects, system stability during the nebulization and aerosol deposition in human airways, which comprise a complex approach using relatively rare methods. According to the findings of the study, such complex testing is necessary for correct prediction of the drug delivery efficacy.

## Supporting Information Available

Detailed view of liposomal vesicle stability during the nebulization by all the investigated nebulizers; charts showing the vesicle size distributions of the investigated liposomal systems before and after the nebulization and the influence of the nebulization process on specific vesicle size (PDF)
