# Supplementary material for: Nebulization and In Vitro Upper Airway Deposition of Liposomal Carrier Systems
Source: Mol Pharm. 2024 Mar 11;21(4):1848–60. doi: 10.1021/acs.molpharmaceut.3c01146 (PMC10988550; doi:10.1021/acs.molpharmaceut.3c01146)
Supplement: Supplementary file 1 — mp3c01146_si_001.pdf [file mp3c01146_si_001.pdf]

Nebulization and in vitro upper airway deposition of liposomal carrier systems

Ondrej Mišík[1], Jana Kejíková[2], Ondřej Cejpek[1], Milan Malý[1], Adam Jugl[2], Miloslav Bělka[1], Filip Mravec[2], František Lízal[1]*

[1]Department of Thermodynamics and Environmental Engineering, Faculty of Mechanical Engineering, Brno University of Technology, Technicka 2896/2, 616 69 Brno, Czech Republic

[2]Institute of Physical and Applied Chemistry, Faculty of Chemistry, Brno University of Technology, Purkyňova 464/118, Královo Pole, 612 00 Brno, Czech Republic

Supplementary data

The vesicle size distributions of DPPC-PEG-Chol and DPPC-PA-Chol measured before and after the nebulization provide an image of the liposomal system damage caused by the nebulization process. These distributions are shown in followed *Figures 1* and *2*.

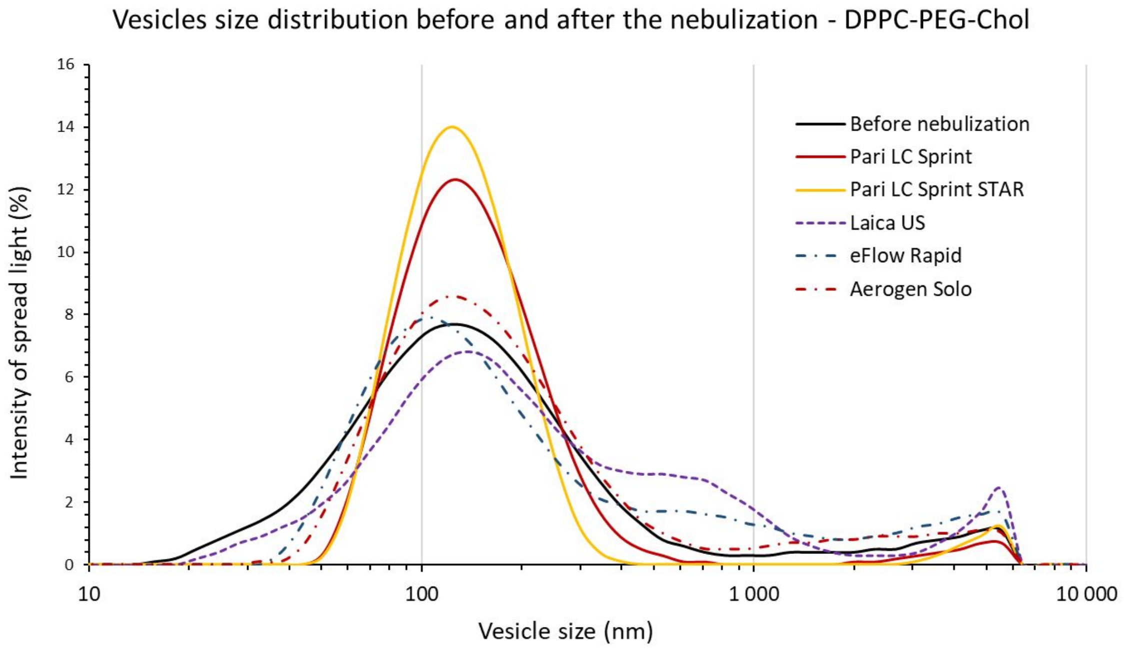

Figure 1: Vesicle size distribution of liposome DPPC-PEG-Chol before and after the nebulization by various nebulizers.

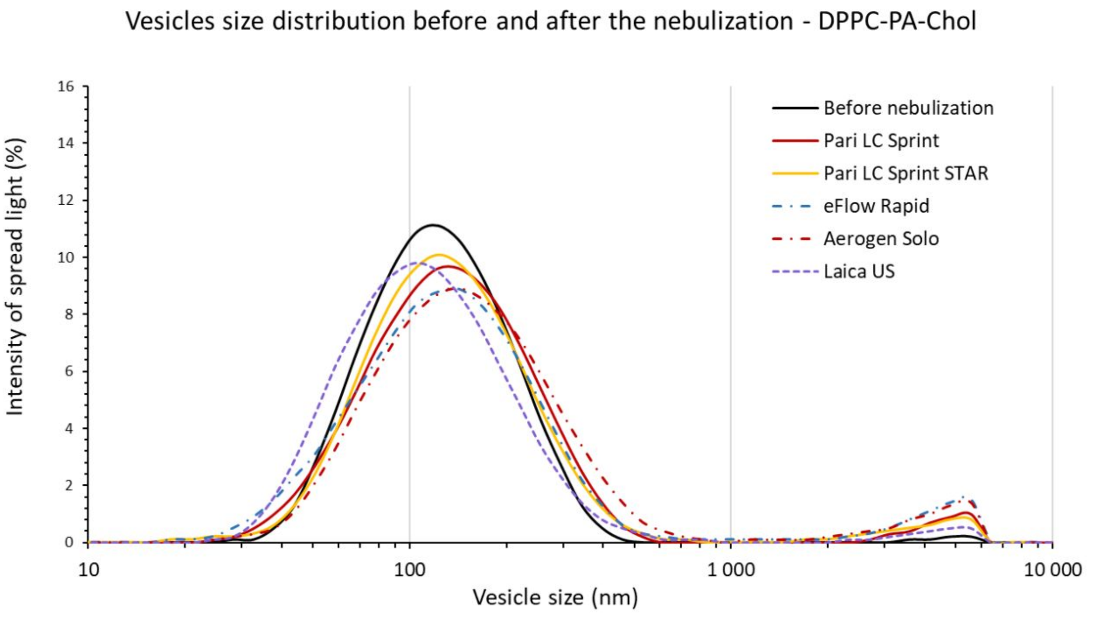

Figure 2: Vesicle size distribution of liposome DPPC-PA-Chol before and after the nebulization by various nebulizers
